# Can technology optimise the pre-operative pathway for elective hip and knee replacement surgery: a qualitative study

**DOI:** 10.1186/s13741-020-00166-0

**Published:** 2020-11-16

**Authors:** Faraz Sharif, Ammar Rahman, Emma Tonner, Hanad Ahmed, Iqraa Haq, Rami Abbass, Shad Asinger, Magda Sbai

**Affiliations:** 1 Imperial College London, Faculty of Medicine, South Kensington, London, SW7 2BU UK; 2 University of Leeds, Leeds, UK; 3 University of Southampton, England, UK; 4 Guy’s and St Thomas NHS Trust, London, UK

**Keywords:** Healthcare professionals, E-health, Mobile health, Virtual reality, Remote monitoring, Telemedicine, Telehealth, Pre-operative pathway, Elective orthopaedic surgery, Qualitative

## Abstract

**Background:**

An ageing population has resulted in a rise in the number of hip and knee replacement surgeries in the UK. The pre-operative pathway is plagued with issues causing long delays and cancellations. Virtual healthcare technologies have a growing evidence base to help solve these issues. One problem of implementing these technologies is the resistance to change mentality from healthcare professionals. By getting their opinions on the place of these technologies within the pre-operative pathway, a united front can be formed to help deliver change.

**Methods:**

Sixteen semi-structured interviews were conducted with key stakeholders within the orthopaedic pre-operative pathway at Imperial College Healthcare NHS Trust. General topics included the different technologies that could be used within the pathway, their uses and associated benefits and problems. Interviews were audio-recorded, before being manually transcribed and then analysed to form categories and themes.

**Results:**

Various uses, benefits and problems were identified by healthcare professionals for each modality of technology. E-forms were seen as a high reward, low-risk intervention. Remote patient monitoring and teleconsultations had their bonuses, but feasibility was a primary concern. Web-based interventions were seen as an intervention of the past, whereas virtual reality was seen as perhaps being ahead of its time. M-health was very positively viewed due to its all-encompassing nature. Digital illiteracy emerged as a consistent problem for most technologies.

**Conclusions:**

Current literature, the results from this study and technology trends within society highlight both M-health and E-forms as the 2 most promising virtual healthcare technologies for use in the pre-operative pathway for orthopaedics. Areas such as pre-operative assessment, triaging and prehabilitation are prime candidates for virtual intervention. Future research should also consider including patient opinions on any proposed interventions, as well as taking into account barriers to implementation.

**Supplementary Information:**

**Supplementary Information** accompanies this paper at 10.1186/s13741-020-00166-0.

## Background

The National Health Service (NHS) faces an ageing population (Office for National Statistics 2018), influencing the variety, frequency and subsequent management of conditions which present to hospitals (World Health Organization (WHO) 2011; Valdes and Stocks 2018). Coined the ‘operation of the century’ (Learmonth et al. 2007), hip and knee replacement surgeries have risen significantly by 90% over the last 15 years (Thompson 2017), now constituting 2 of the 3 most performed surgeries in the NHS (Health and Social Care Information Centre 2013).

However, the pathway for this procedure is by no means ideal with patients facing long waiting times and last-minute cancellations (Briggs 2015; Torjesen 2018). The reasons for cancellations are multi-fold, but the majority have been attributed to inadequate triaging, preparation and optimisation in the pre-operative process (Torjesen 2018; Moonesinghe et al. 2013). This costs the NHS an estimated £400 million in operating time lost each year (Gillies et al. 2018).

Technological advancements have been highlighted by various policy documents to hold the answer to effectively managing cost burden and resource allocation within the healthcare system (NHS England 2014; Health Education England 2019; Wachter 2016). It is estimated that deployment of technological solutions could save the NHS an estimated £2.9 billion (Ofcom 2013; PricewaterhouseCoopers LL 2013). This has been supported by a growing research base; several trials in the UK and globally have shown varying types of technologies, hereafter referred to as virtual healthcare technologies (VHT), delivering increased patient satisfaction, treatment adherence, cost-savings and more efficient use of patient and clinician time (McCue et al. 1997; Robinson et al. 2017; Morrice et al. 2019; Gill 2011).

VHT can be defined in the literature, as information technology that deliver remote interactions between patients and/or healthcare professionals (HCPs) to facilitate patient care (Jamieson et al. 2015). A systematic review of the existing literature showed the following VHT being used in different capacities in the orthopaedic pre-operative pathway: electronic forms (E-forms), websites delivering written information, websites delivering online videos, teleconsultations, remote patient monitoring (RPM), virtual reality (VR) and mobile health (M-health).

The literature review identified several gaps in the evidence base. Firstly, current literature is heavily focussed on the benefits of the technologies, failing to comprehensively evaluate limitations in practice. Secondly, most interventions were conducted outside of the UK. Hence, their feasibility and impact to the NHS must be critically analysed. Finally, there is a lack of insight into the opinions of HCPs on VHT. With resistance to change from the workforce seen as a primary barrier to adopting new practice (NIfC E 2007), it is essential to explore the views of these key stakeholders in order to deliver change. Therefore, our study will collect a range of opinions to provide a more balanced view of VHT from the key stakeholders within the NHS.

## Methods

### Study design

A qualitative method of data collection was employed, using semi-structured interviews (SSI) to speak to HCPs. The use of SSIs allowed deeper exploration of thoughts, attitudes and beliefs. Additionally, SSIs allowed comparability between interviewees whilst avoiding undue influence from other stakeholders in the answers provided (Barriball and While 1994).

### Settings and participants

Using a process map created in collaboration with senior members of the trust, key stakeholders involved in the pre-operative pathway for elective hip and knee surgery were selected for interviews. This ensured an even level of participation across the pathway, giving this study an appropriate level of scope as well as depth of research. Sixteen interviews were conducted with a range of HCPs, including 2 general practitioners (GP), 2 orthopaedic surgeons, 2 anaesthetists, 3 orthogeriatricians, 3 nurses, 2 occupational therapists (OT) and 2 physiotherapists.

As the pre-operative process varies from trust to trust, participants were limited to Imperial College NHS Healthcare Trust to ensure consistency.

Participants were contacted via email and interviewed in-person (one interview conducted via Skype) in a private setting.

### Interview guide

The interview questions (Additional file 1) were formulated based on a systematic review of the literature that identified the key VHTs. They followed an inductive method to explore their uses, benefits and problems from a stakeholder perspective. Questions to corroborate the process map were also included. The SSI style permitted themes not contained in the guide to arise and be investigated.

The interview guide was pilot tested with 4 HCPs before use.

### Data collection and analysis

Two interviewers conducted each interview; interviewer one asked the core questions whilst interviewer two wrote notes with any follow-up questions asked that may have been missed. All interviews were conducted by the same interviewers.

All interviews were recorded and stored on a secured OneDrive account, in accordance with the Ethics regulations of the project.

Interviews were manually transcribed, with identifiable information removed from the transcripts. A thematic analysis (TA) was subsequently conducted, using the six-step process outlined by Braun and Clarke (Braun et al. 2019). At each stage, the work was cross-checked by other team members to eliminate errors. Coding was conducted alongside the interviews to identify when data saturation was reached and when interviews could be stopped (Guest et al. 2006).

### Ethics

Ethical approval was received from Imperial College Research Ethics Committee on 5/2/2019.

## Results

In the 16 interviews conducted with HCPs, 7 modalities of virtual technology were discussed: teleconsultations, web-based written information, web-based online videos, E-forms, RPM, VR and M-health. The results of the TA are categorised under each modality, with their associated uses, benefits and problems in the pre-operative pathway for elective hip and knee replacement surgery.

**Fig. 1:**
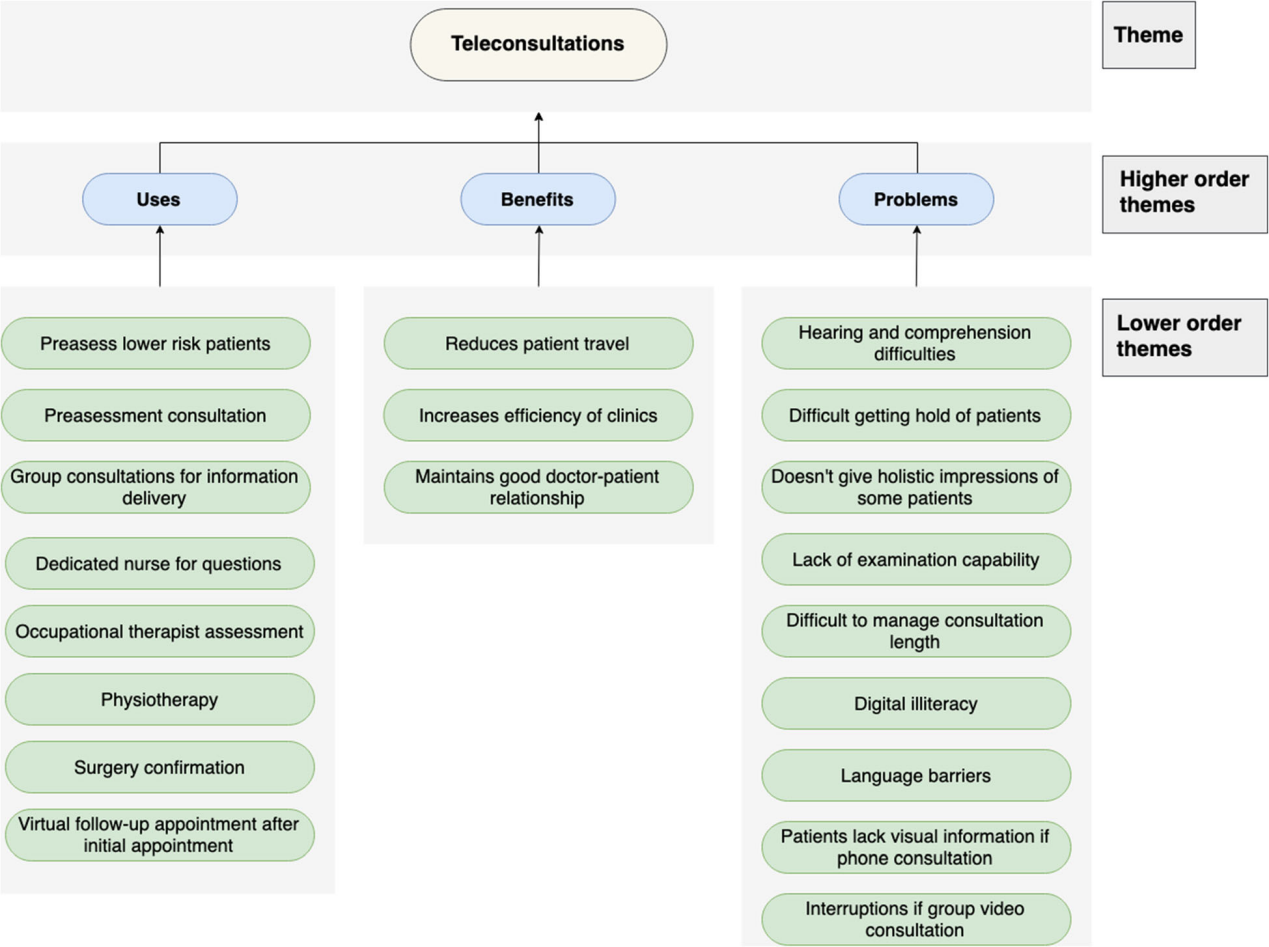
Chart displaying the use-cases, benefit and problems of teleconsultations

In the following tables, lower order themes have been listed in descending order of the number of interviews they were suggested in (Fig. 1).

### Teleconsultations

#### Use-cases

Teleconsultations were highlighted useful for the ‘pre-assessment clinic’, especially for lower risk, ‘fit (and) healthy patients who don't need to come in’. Nurses can assess by asking questions ‘over the phone’ in conjunction with the patient’s medical history obtained from their GP. Another opportunity highlighted was physiotherapy and OT appointments for teleconsultation pre-assessment; the former able to provide ongoing support for their patients both ‘pre and post-surgery’ whilst it would be “perfect” for the latter to obtain key information sooner to allow ‘time to plan [for discharge]’. Information and reminders as well as questions and virtual follow-ups could be delivered via teleconsultations, as a means of communication between HCPs and patients. The information delivery could be one-to-one or via group sessions which could be ‘more efficient’ so the patient does not have to ‘waste time and money on transport’.

#### Benefits

Teleconsultations reduce patient travel. This is particularly helpful for the elderly who may struggle more with transportation. In addition, a teleconsultation could allow the maintenance of a doctor-patient relationship, with video interaction allowing you to speak to and reassure patients, but also increasing the efficiency of running clinics. It helps to ‘better utilise the small services that we have got’ and shortens the face-to-face consultation time by ‘deciding what tests and investigations need to be done from a teleconference type conversation’ before the appointment.

#### Problems

Teleconsultation may be difficult if there are hearing or comprehension difficulties and could prevent a successful consultation. Further problems arise for ‘those that do not have English as a first language’.

There is a lack of visual information compared to ‘when you’re in the hospital...it gives you an opportunity to see things’. Additionally, doctors would not be able to examine the patient. Doctors stated that it is useful to ‘get an overall impression of them. How are they walking? How are they interacting?’

**Fig. 2:**
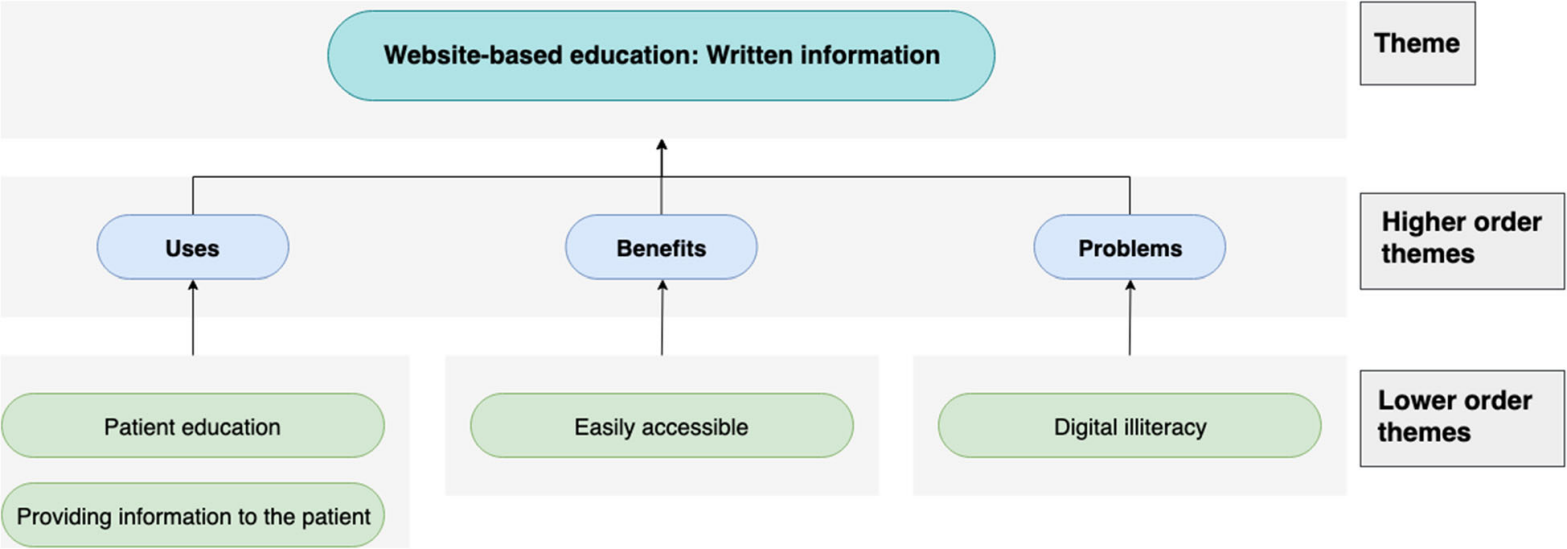
Chart displaying the use-cases, benefit and problems of web-based written information

It may be difficult to get hold of a patient due to incorrect personal details or incoherent timings. Moreover, once you have started the consultation, it may be difficult to manage the consultation length and ‘try and stop them’. There is also the risk of being interrupted by external events, ‘random people could come in’ (Fig. 2).

### Web-based written information

#### Use-cases

A website can be used as a platform to share information with patients such as ‘what to expect’, ‘what you need to do next or who you should contact’ and ‘various exercises for patients to follow before they have their surgery’. This is a good resource ‘to equip people with as much information prior to their surgery so that they make an informed decision’.

#### Benefits

A website is ‘accessible to everybody’, as both patients and their families would be able to read the information. It is also considered more user-friendly because ‘of the ages of the patients’.

#### Problems

**Fig. 3:**
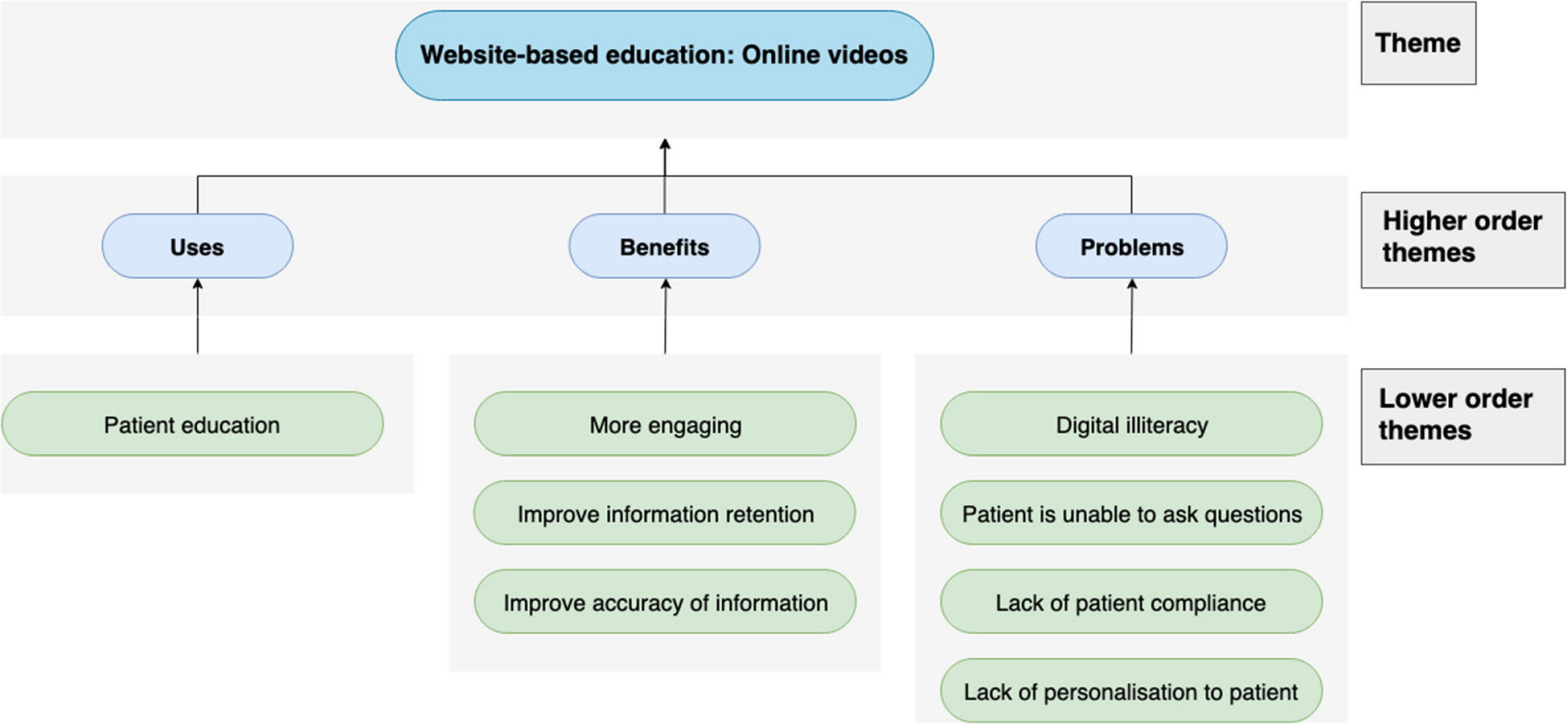
Chart displaying the use-cases, benefit and problems of web-based online videos

There is a concern surrounding ‘computer [digital] literacy [and] whether the patients will [be able to] access them’ (Fig. 3).

### Web-based online videos

#### Use-cases

The sole use identified for online videos is patient education, as patients would benefit from a ‘video of what to expect at the appointment’. The videos could include a walkthrough of the wards; interviews with the matrons and what their day-to-day activities would involve. Videos can also improve patient preparedness for appointments as ‘they’ll come with their medication list’ which ‘will be quicker, more streamlined’. Finally, videos can act as an adjunct to joint school education as patients can re-watch anything missed, or it may benefit non-English speaking patients through translations**.**

#### Benefits

Patient engagement could rise as it would ‘condense a lot more [information] into a shorter space overall’ with patients preferring a 10-min video to reading through 10 pages of text. Additionally, because many patients are visual learners, video content could benefit them more. The modality may also aid in information retention since the videos can be watched several times and the accuracy of information can be assured with the videos scripted by healthcare professionals.

#### Problems

**Fig. 4:**
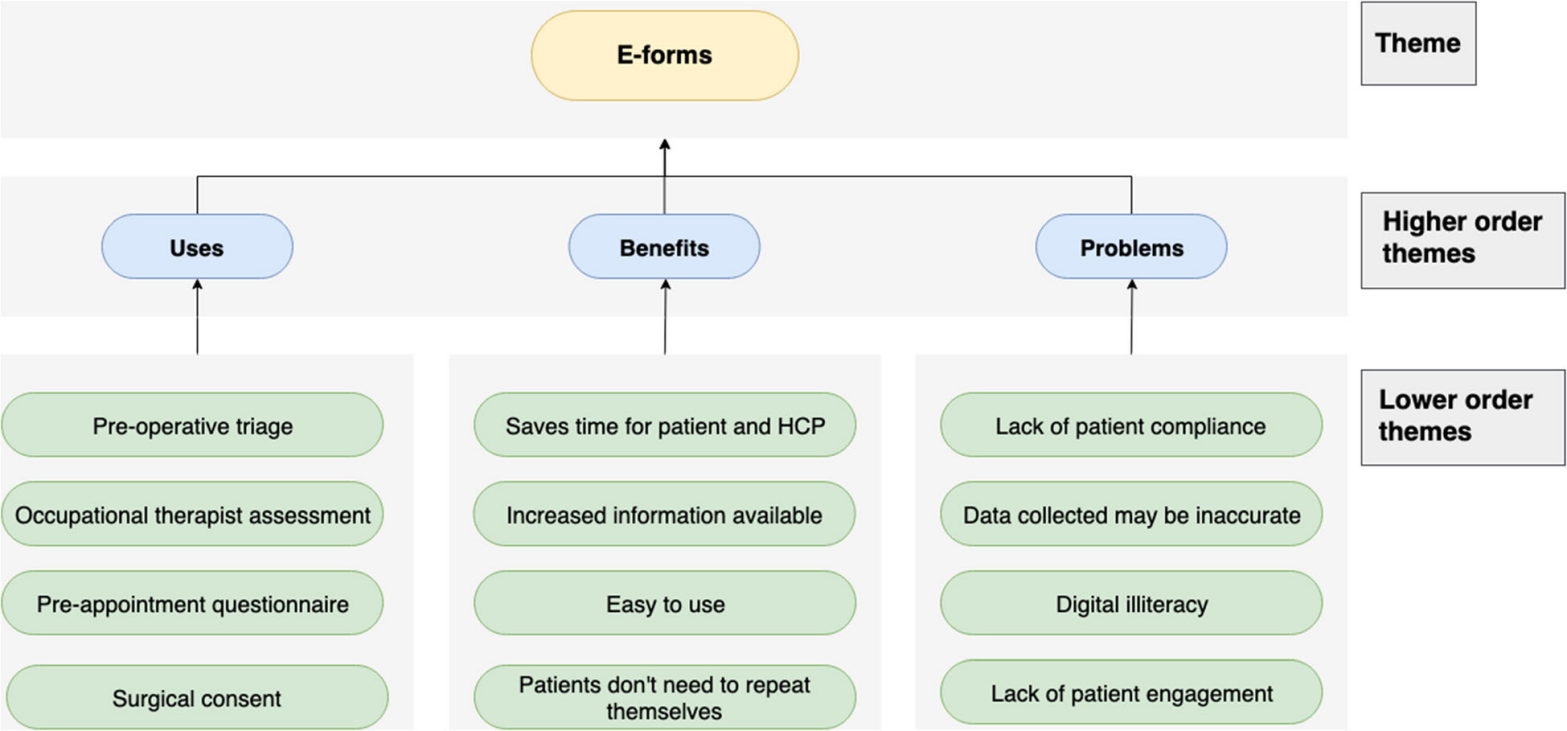
Chart displaying the use-cases, benefit and problems of e-forms

As the solution needs to be targeted at an older demographic, there are concerns regarding digital illiteracy. Online solutions remove the need for face-to-face education, which means ‘if it was purely done on a video, people wouldn't have the opportunity to ask questions’. A virtual solution could also pose as a challenge for compliance, as ‘you still have the challenge of whether the patient actually read the electronic leaflets’, an issue shared with online videos. Finally, standardized online videos to achieve patient-centred and individualised care could be difficult (Fig. 4).

### E-forms

#### Use-cases

E-forms could be used as part of OT assessment; using a ‘self-assessment questionnaire’, patients could submit information virtually so that ‘you can have the person and their information in front of you’, at their consultation. A lot of the necessary information including ‘furniture heights, the hip precaution information can be provided’ beforehand virtually. Patients often fill in questionnaires on arrival; ‘medical history that could have been all sorted out in advance’ using a virtual form, saving time in the clinic and directly inputting information into the IT systems. Any unclear information can be clarified on a call. Getting this information in advance can also identify high- and low-risk patients and thus, help plan for suitable appointments. Consent forms could also be virtualised.

#### Benefits

E-forms ‘prevent people coming into appointment and then being asked the same question over and over again’. It saves nurse’s time in the pre-operative appointments, which allows patients to ‘actually talk about their problem more’. It can ‘easily be done as a screening tool at home without the patient having to come to see us [the HCP]’, saving ‘a lot of wasted journeys for patients’.

#### Problems

**Fig. 5:**
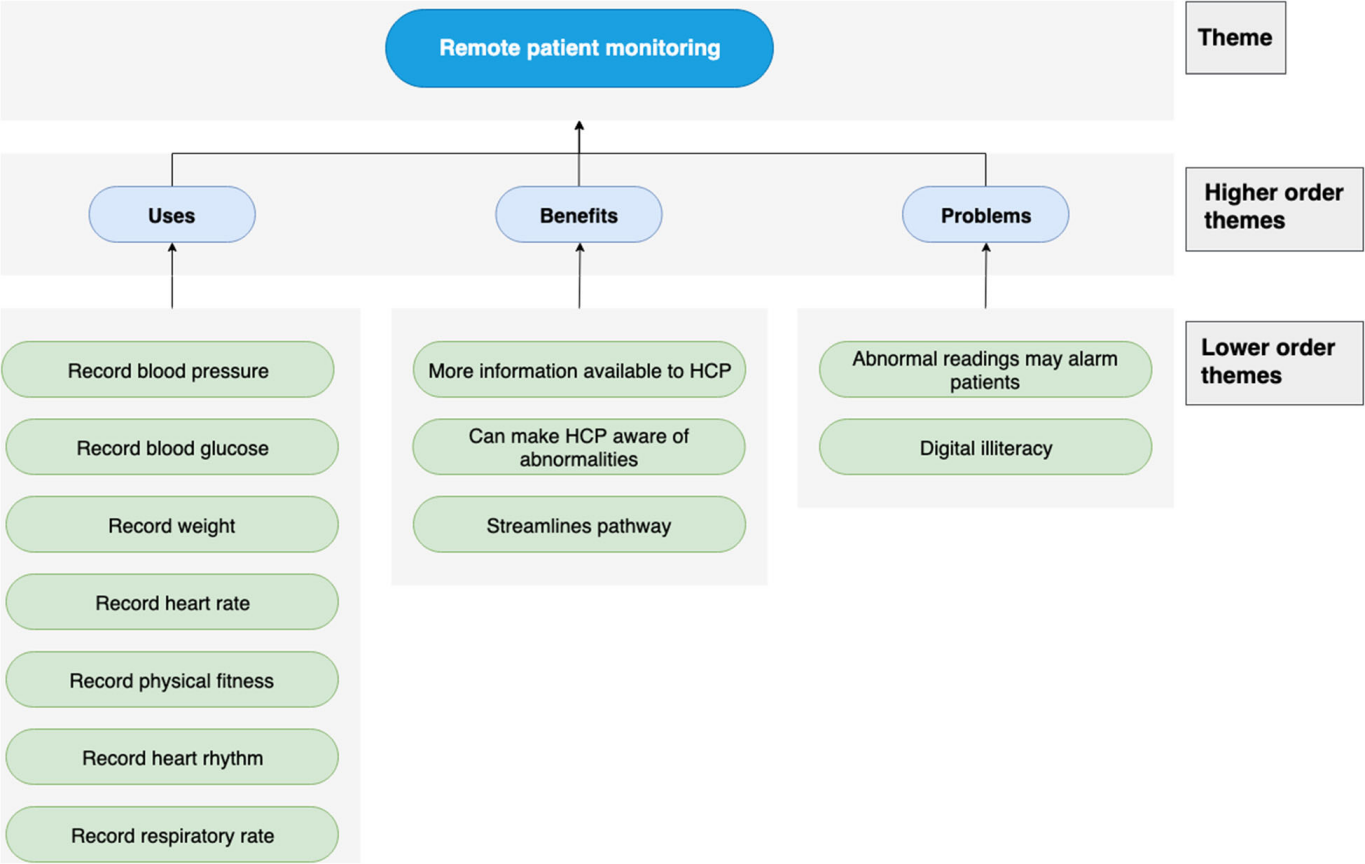
Chart displaying the use-cases, benefit and problems of remote patient monitoring

There may be difficulties for patients filling in the questionnaire, leading to ‘blank’ and missing information as they may misunderstand the questions. However, if it is mandatory, patients must either call for clarification or may mistakenly provide false information. Filling in the forms requires the patient to be ‘honest’, yet, patients are more likely to be open in face-to-face consultations. For it to succeed, they need to be engaged and compliant: ‘There might be some patients who would find it difficult or would not want to engage in that way or would need to ask someone else to do it for them’. Finally, patients may either not have access to the digital technology or they are ‘not [digitally] literate’ enough to engage (Fig. 5).

### Remote patient monitoring

#### Use-cases

Numerous parameters were identified that could be measured and recorded remotely, including blood pressure, blood glucose, weight, physical fitness, heart rate, heart rhythm and respiratory rate. Due to the widespread use of smartphones, HCPs can track changes and progress in a patient’s condition as improvement in physical activity can be reassuring. It can also monitor whether they are meeting their recommended daily exercise goals.

#### Benefits

RPM provides HCPs with greater information to assist in management. For example, a patient may present with high blood pressure in hospital which could be attributed to ‘white coat syndrome’ whilst at home, their blood pressure may be normal. Greater availability of information also helps detects abnormalities that can be followed up by the preoperative team.

#### Problems

**Fig. 6:**
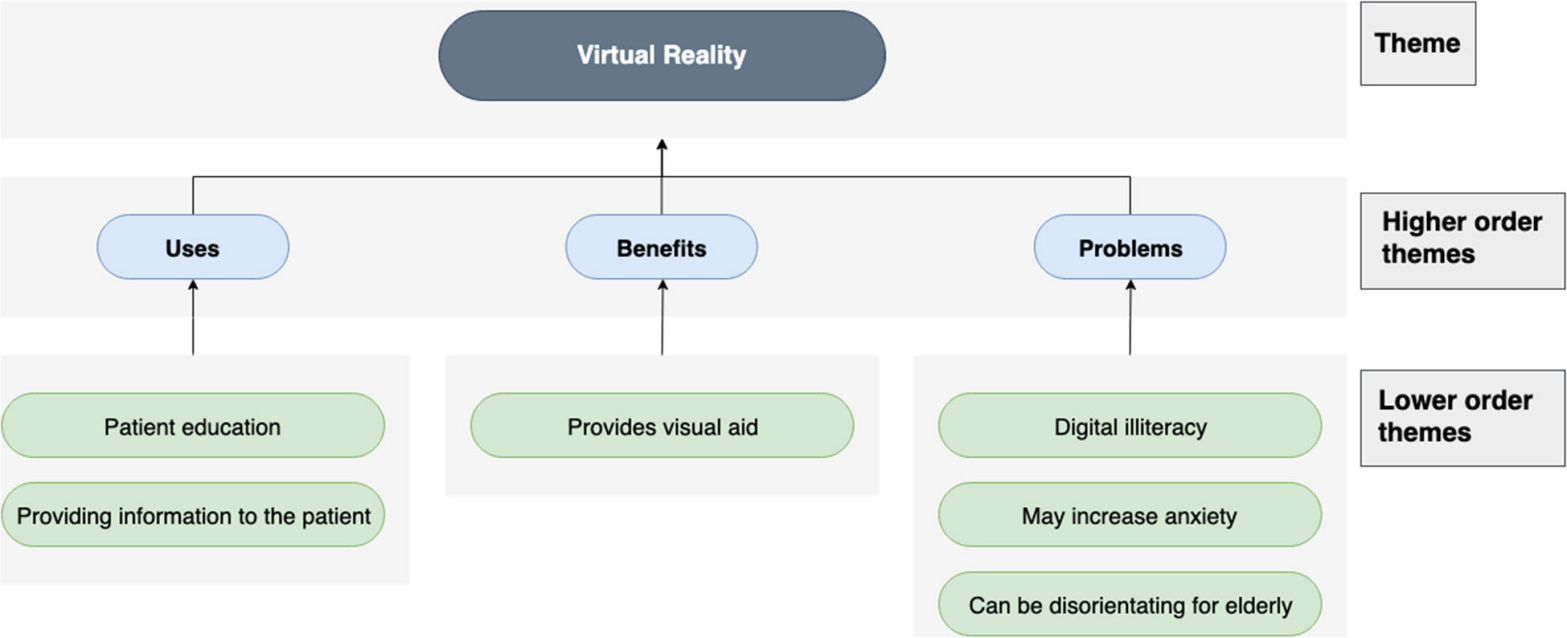
Chart displaying the use-cases, benefit and problems of virtual reality

By allowing patients to record their own readings, there may be higher incidences of anxiety if patients see ‘error messages or abnormal data’. Finally, patients would need to be able to operate the equipment, ‘it should be fairly self-explanatory, but not everyone is tech savvy’, and so the equipment could be ineffective if patients cannot successfully use it (Fig. 6).

### Virtual reality

#### Use-cases

VR can be used for patient education and information delivery. Patients could be given a visual representation of their pathway: ‘how it will look in the theatre room’ for example, that they would not normally be aware of. It can be used to explain difficult conditions, i.e. ‘maybe you could replicate delirium, or you might be able to replicate it for family members’ so that patients and families are better prepared, to increase awareness and insight.

#### Benefits

Using VR, patients would be able to visualise their experience rather than ‘having a verbal explanation or a written leaflet’. This is equivalent to ‘almost giving an experience without having to having to do it’, which can make it ‘not as scary’.

#### Problems

**Fig. 7:**
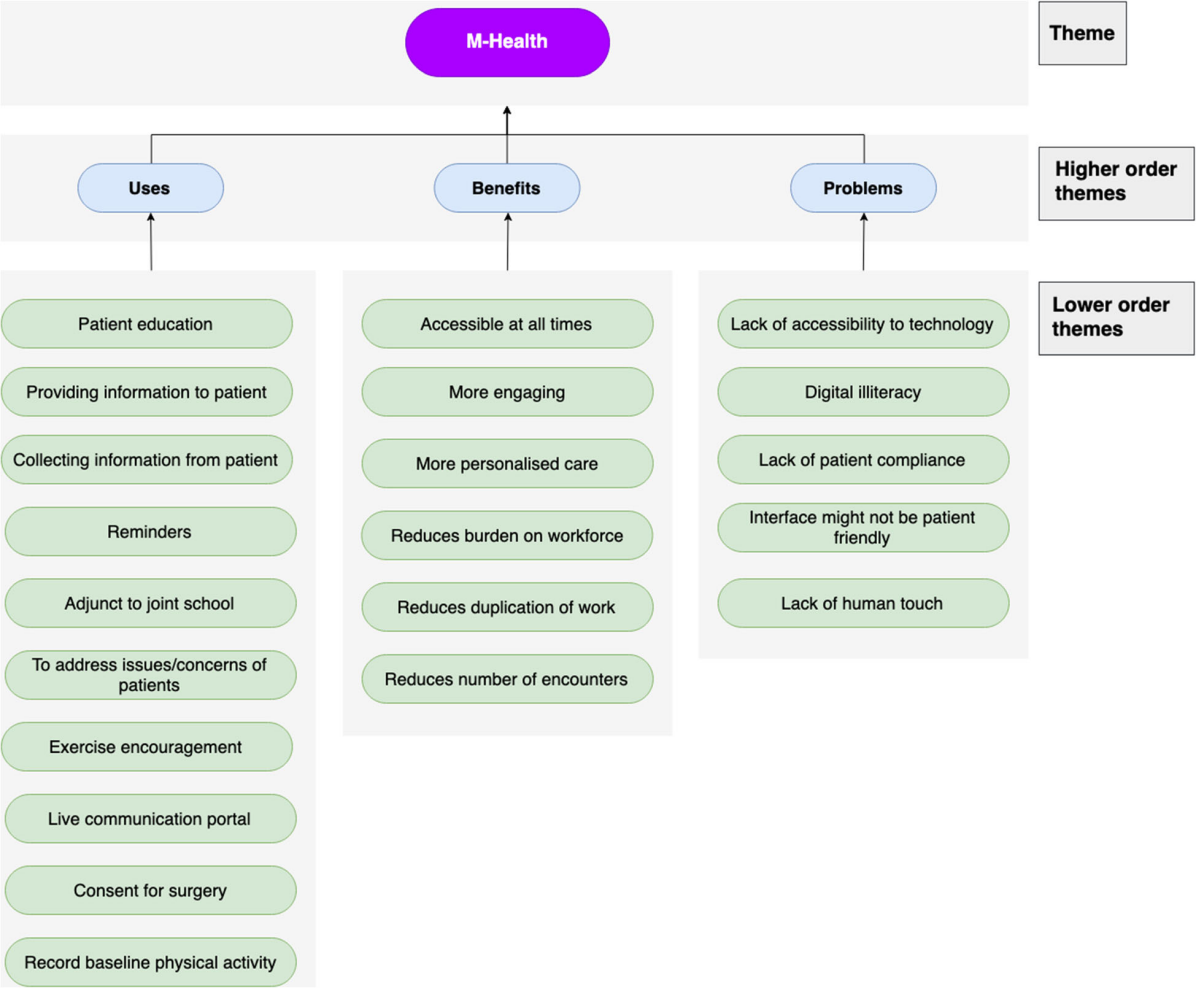
Chart displaying the use-cases, benefit and problems of m-health

Concern was raised about VR: perhaps it is ‘over complicating fairly straightforward things’. It could ‘increase anxiety’ because ‘orthopaedic surgery [is] very scary’ and knowing more may in fact make the patient more apprehensive, rather than alleviating their concerns. It may also be disorientating for the elderly: ‘My partner gave it [VR device] to his grandfather, and he nearly fell over because if you take somebody visuals out, if they’re relying on that for their balance’ (Fig. 7).

### M-health

#### Use-cases

M-health can be used to provide a range of information; it can answer ‘frequently asked questions and eventualities and you can see your journey pathway’. This can help reduce anxiety surrounding surgery and make ‘it very clear what their preoperative preparation time should be, how long they should be starved for, when they should come in, where they should go...’. It is also a ‘very good way of reinforcing learning’, including pre-operative physiotherapy exercises. Baseline physical information including exercise and pain can be recorded, and it can be used to encourage an increase in activity before surgery.

M-health can be used to provide important reminders such as what patients need to do in preparation for surgery and give live updates of appointment times on the day. It can also be used in ‘letting them know their investigation results’. For the HCPs, it can be used to collect information from the patient in the form of questionnaires and consent for the surgery. There could also be a ‘two-way communication’ channel providing a ‘gateway for them [patients] to be able to ask questions back to the surgeons’, helping to reassure them and alleviate anxiety.

#### Benefits

M-health is beneficial because ‘it’s accessible to someone all the time’. It is helpful to clinicians as it could be using to triage and ‘filter[s] out some inappropriate presentations to the unit’. This, alongside collecting information in advance, can ‘reduce the number of encounters necessary to the patient’ where appropriate. M-health applications can also provide more personalised care by adapting to their user. It was suggested that M-health questionnaires are more engaging for patients and that they help ‘improve the interface for [questionnaires]’.

#### Problems

Problems with M-health centre around its accessibility and use. Not all ‘patients have smartphones and tablets’ and those who do, may need somebody to help support them using and understanding it, particularly elderly patients who may also struggle logging on and remembering their passwords. It must be easy to use with a friendly interface suitable for ‘people that don’t have great fine motor dexterity or visual issues’. The number of available Apps also added to patient’s confusion. Patients need to use the application and fully engage with it; it is easy for patients to use technology as an excuse or choose not to use it, and difficult to monitor their engagement with it. There is also a risk that ‘Once you replace humans then you lose the interaction, you lose the feel [human touch]’.

## Discussion

To help determine the most beneficial VHTs and their relative impact in the pre-operative pathway, it is necessary to compare the results of the TA with the existing literature. These will be discussed and categorised according to VHT modality.

### Teleconsultations

Teleconsultations were placed as most useful in the pre-operative assessment clinic, with the greatest benefit being reduced travel and cost savings for patients. Clinicians were particularly satisfied with this benefit as it does not compromise face-to-face contact. The TA also indicated that staff would find tele-consultations helpful in identifying high-risk patients aiding earlier triaging and optimisation, potentially reducing last minute cancellations. This is supported by Tam et al. whose use of teleconsultations resulted in a drop in cancellation rates from 10 to 3.1% (Tam et al. 2017). Our TA also highlighted that tele-consultations prior to clinic appointments can inform nurses of which tests need to be organised for each patient, reducing waiting times and delays for both patients and staff. Both these suggestions are supported by the literature and utilise important demand management concepts of ‘filter and focus’, triaging patients according to risk and need, allowing for demand forecasting and optimal resource allocation. By optimising the ‘back office’ support services that organise and administrate patients, clinics can be run with minimal variation and on schedule, reducing patient waiting times.

Another important concept identified was the use of teleconsultations in the continuous monitoring of patients throughout the pre-operative pathway. HCPs felt that virtual follow-ups between appointments would be beneficial to patients that are unsure about whether to report any changes in their health or wellbeing. This suggestion mirrors the case report by Blozik et al. (Blozik et al. 2012), showing the effectiveness of tele-consultations in avoiding last minute cancellations and post-operative complications by providing a contact point between appointments and before their surgery. By staying ‘in touch’ with the patient, the organisation takes responsibility for the entire care cycle, improving patient experience and outcomes, whilst reducing costs incurred by complications and cancellations (Porter and Lee 2013).

The TA highlights important barriers to the use of teleconsultations in clinical practice. One major barrier is the inability to physically examine patients. Although literature shows high levels of concordance between virtual and face-to-face examination, these examinations were only relevant to the anaesthetic context and did not consider orthopaedic examinations and more extensive tests (Applegate et al. 2013). Furthermore, clinical staff also expressed the importance of seeing patients in person, to implicitly judge their physiological reserve and general condition. The TA also indicated a use for tele-consultations in the daily workflow of OTs, although there is no supporting literature for this use-case.

Despite great promise, the limitations in physically interacting with patients as well as concerns about scheduling alongside current appointment systems makes implementing tele-consultations an extremely challenging task. HCPs also discuss alternative, less challenging technology modalities able to fulfil the benefits previously discussed. This, coupled with a lack of data economically evaluating tele-consultations, means that whether they are the answer therefore remains unascertained.

### Website-based education: written information

The only use identified for this technology was patient education. A lack of interviewee enthusiasm resulted in a lack of data on this topic, perhaps due to how well-established this technology is.

HCPs interviewed described the main benefit of websites as easy access. Literature further demonstrates that information-based websites are a more effective means of delivering information, resulting in a better-informed patient (Heikkinen et al. 2008), increased patient satisfaction and reduced anxiety (Yin et al. 2015).

Interviewees cited digital illiteracy as the largest problem with this technology, confirmed by Yin et al. (Roughead et al. 2016). Additionally, literature shows that website-based information needs to be updated on a regular basis, particularly as there is often unverified or inaccurate information available online (Roughead et al. 2016).

### Website-based education: online videos

HCPs identified patient education as the sole use for online videos. This concept has been previously tested, where it demonstrated a reduction in pre-operative anxiety, whilst better preparing patients (Roughead et al. 2016). HCPs concurred with this, identifying online videos as more engaging and increasing information retention. HCPs also pointed out that they could ensure information accuracy by producing the video themselves.

However, the aforementioned study failed to identify problems for online videos. The interviewees provided greater depth illustrating how digital illiteracy would be a roadblock. They also expressed concerns that the patient would be unable to ask questions, the videos would not be tailored to patients and they could not guarantee that patients watched the videos.

### E-forms

Waller et al. (O'Connor et al. 2016) discussed using E-forms to collect information from patients prior to their consultation. This correlated with the HCPs who suggested this be done at multiple stages of the pathway including for the surgeon, the nurse and the OT. They also suggested using information collected via E-forms to risk-stratify the patient earlier. In addition, one HCP wanted surgical consent to be taken via this method as well. This use has evidence base behind it, as Issa et al. (Waller et al. 2015) showed how electronic consent was preferred by patients whilst being more standardized, easier to read and comprehensible. More recently, St John et al. (Issa et al. 2006) demonstrated their use at separate hospitals in the UK in various departments and that electronic consent improves quality and consistency of documentation.

The interviewees cited saved time and reduced duplication of information as the key benefits of E-forms. They enjoyed the ease of use of E-forms and appreciated the impact it could have on increasing continuity of care. This is supported by Staroselsky et al. (St John et al. 2017), who suggested that getting patients involved in reviewing and submitting their own health information could result in a more complete EHR. Literature also suggests that collecting information would provide a more holistic view of the patient (O'Connor et al. 2016).

HCPs suggested digital illiteracy as a potential barrier. However, they stressed access to technology is rising across age groups and could be improved with adequate support. This is supported by a study from Deloitte which showed that 77% of over 55s own smartphones, with this number expected to continue rising (Staroselsky et al. 2006). The interviewees also suggested that data collected may be inaccurate and patient compliance may be an issue.

### Remote patient monitoring

A comparatively newer technology, HCPs supported the use of RPM in monitoring patient prehabilitation by measuring physical activity. Darvall et al previously illustrated this by incorporating pedometers (Adams and Lee 2019). However, they noted that a major limitation was that walking may not be possible for people about to undergo knee or hip replacements (Adams and Lee 2019). Interviewees further suggested that RPM could play a bigger role in the pre-operative pathway in monitoring a patient’s health prior to surgery (e.g. blood pressure and blood glucose).

HCPs also had positive impressions of the modality, noting that having constant RPM would increase the pool of information available to them, saving time collecting information whilst allowing more informed decisions. RPM could also alert HCPs to any emergency abnormalities. Furthermore, a major study run by the UK government showed that home-based telemonitoring devices delivered a reduction in accident and emergency (A&E) visits, a drastic 45% reduction in mortality rates, as well as an 8% reduction in tariff costs (Darvall et al. 2016; Department of Health 2011).

The problems with RPM make its feasibility questionable. The aforementioned UK government trial showed high implementation costs of RPM with cost savings that were statistically insignificant (Darvall et al. 2016; Department of Health 2011). As well as this, the interviewees also identified the potential of medicolegal issues regarding data collected. There was the possibility also that the patient may be unnecessarily alarmed by abnormal but insignificant readings. Again, digital illiteracy was acknowledged as a potential barrier.

### Virtual reality

The sole application of VR in the pre-operative pathway for elective orthopaedics identified by the interviews was educating patients about before, during and after surgery. As demonstrated by Bekelis et al. (Newman et al. 2013), VR used this way improved satisfaction, preparedness levels and decreased anxiety.

Interviewees, however, were more sceptical about this technology, particularly in the elderly demographic. They highlighted that the elderly may struggle with this futuristic technology and may find it disorientating. They also had concerns that VR could even increase anxiety for some patients by over-burdening them with information.

### M-health

M-health was one of the most popular modalities amongst HCPs interviewed. M-health encompasses all the aforementioned technologies to some extent and could be incorporated into an M-health solution. Interviewees suggested the use of m-health to monitor patient adherence to pre-operative protocols outside of hospital, a use supported by research (Bekelis et al. 2017). They proposed using M-health to give patients instructions and reminders regarding their pre-operative preparation. They further advocated a larger role for M-health in other parts of the pathway including patient education, pre-appointment questionnaires, as a means of communication as well as for RPM.

The interviews agreed with literature that M-health could help reduce duplication of information and reduce the workload of HCPs, particularly if the application was linked to EHRs (Bekelis et al. 2017). However, HCPs added that there were multiple additional benefits of M-health, including its increased accessibility for the patient, the fact that it is more engaging whilst also providing more personalised care.

**Fig. 8:**
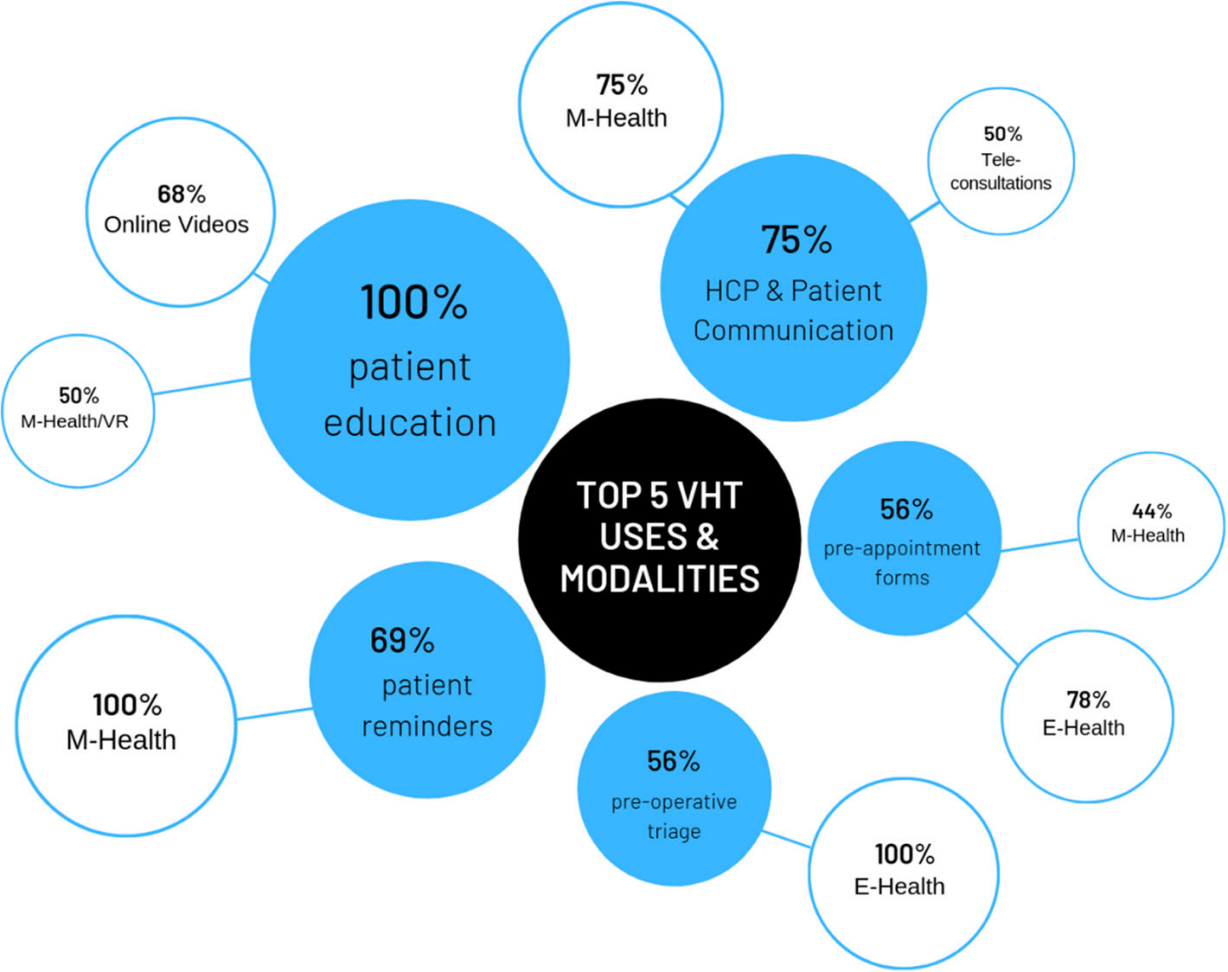
Top 5 uses of VHT suggested by HCPs in TA alongside preferred mode of delivery. Percentages illustrate the number of healthcare professionals that made a suggestion

From the interviews, it was discussed that M-health intervention could present problems as people may not have access to technology or may not know how to use it. In addition, they stated it would be difficult to get patients to comply with the application. The last point is in direct contrast to the study by Kim et al. (Bekelis et al. 2017), who showed that adherence rates for M-health were similar and could be improved further if the benefits of the application were evident to the patient (Fig. 8).

## Implications to practice

The TA shows what HCPs think the best uses of VHT are and which modality they would most like to see delivering these. The results of the top five uses and their preferred associated modalities are shown in Fig. 8.

A series of audits by the Royal College of Anaesthetists (Royal College of Anaesthetists 2019) showcases two key problems that could be tackled by these virtual interventions:

1. Triage and pre-assessment
2. Patient education and preparation

Hence, we propose the following recommendations.

### Recommendations

1. Patient triaging and pre-assessment: Patients should be provided with an interface with access to a virtualised triaging e-form. This would be based on the current triaging guidelines. The triaging tool would collect information on a patient’s demographic, co-morbidities and lifestyle, and automatically triage patients into low, medium and high-risk groups according to the ASA (American Society of Anesthesiologists 2014). This could have a significant impact on workload, with 1 in 6 patients undergoing hip and knee replacement being considered low-risk patients (National Joint Registry 2019). Patients will thus be filtered down the appropriate channel allowing more efficient time and resource allocation. After triage and preassessment, patients should have a communication portal with a nurse from the surgical team which they can use until the day of surgery to get updates or ask have their concerns addressed. This could prevent on-the-day cancellations.
2. Virtualisation of patient education and preparation: Information normally provided during a ‘Joint School’ could be incorporated into an m-health app. Better informed patients lead to better prepared patients, reduced cancellations and improved post-surgical outcomes (Kearney et al. 2011). This would reduce the resource burden, as despite high initial investment costs in building an app, this would be overcome by the time and costs saved from staff repeatedly organising and teaching Joint School. Additionally, prehabilitation could be encouraged by providing animated instructions for pre-operative physiotherapy exercises as well as reminders to perform these exercises daily to improve patient compliance and adherence to these regimes. Exercise encouragement and prehabilitation has been specifically emphasised to improve post-operative outcomes (Wynter-Blyth and Moorthy 2017). Furthermore, supplementary features such as patient forums on the m-health app could provide comfort and mental preparation for the patient, whilst reminders could also be used to advise patients on medication and nutrition in the days leading up to surgery.

**Fig. 9:**
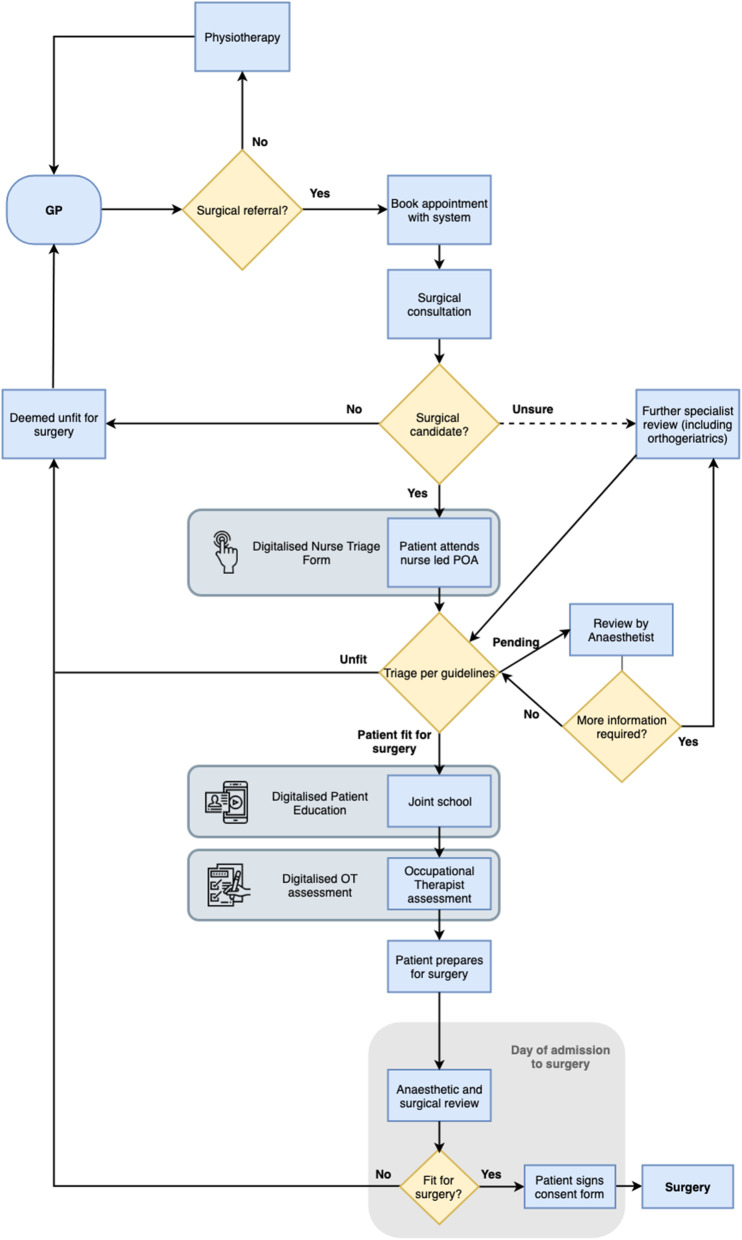
Process map for elective orthopaedic pre-operative pathway at ICHT with proposed recommendations incorporated

These proposed recommendations have been added to the process map for the ICHT orthopaedic pre-operative pathway in Fig. 9 to give added perspective.

### The elderly population and technology

A recurring problem identified throughout the TA and repeated in literature was digital illiteracy in the elderly population (Smith 2013; NHSE 2017). Considering the high average age (69 years) of elective orthopaedic patients, this could be seen as a major flaw of using technology in this space.

However, Deloitte research in 2018 shows 77% Smartphone penetration in the over 55 age group with technology penetration in this age group expected to further rise as it has over the past 6 years (Staroselsky et al. 2006). This makes mobile health and virtual care a promising opportunity for the older patient and their clinicians. In addition, nationwide programmes such as The One Digital programme by Age UK54 are demonstrating the success of working with the elderly to allow them to better engage with technology.

To promote adoption of innovation, it must be user-friendly, addressing the needs of the consumer and provide a tangible benefit to them (Homburg et al. 2009). Numerous studies have also shown that given convenience, perceived benefit and ease of engagement with the technology, the older demographic tend to adopt new technology in line with their younger counterparts (Venkatesh and Davis 2000; Chen and Chan 2011; Heinz et al. 2013).

### Economic implications

With the NHS suffering from a funding gap of £30 billion a year by 2020/215, costs are a significant barrier. Interviews emphasised that any solution must be cost effective with return on investment. Unless the new technology can recoup the original costs efficiently and require minimal maintenance, it is unlikely that the NHS would prioritise it.

The literature provides contrasting opinions on the cost-effectiveness of technology in general healthcare. A systematic review by Mistry et al. (Mistry 2012) demonstrated no conclusive evidence for the cost-effectiveness of telemedicine and telecare. However, more recent reviews by Delgoshaei et al. (Delgoshaei et al. 2017) and Michaud et al. (Michaud et al. 2018) indicated cost savings associated with telemedicine in various medical fields. It must be noted, however, that there was a stark lack of research on the cost effectiveness of technological interventions in the pre-operative pathway.

A 2011 document from the Department of Health modelled a virtualized pre-operative assessment system, showing savings of £65.83 million simply by increasing efficiency in collecting data and reducing the number of cancellations (Online Preoperative Assessment [Internet] 2011). This was at a Rough Order of Magnitude (ROM) cost of £1.7 million over 5 years, that included costs of infrastructure, development and technical support for 5 years (Online Preoperative Assessment [Internet] 2011).

In addition to the possible financial benefits, technology enables more societal and indirect benefits such as decreased hospital stays, improved quality of life and decreased nursing and residential care by allowing patients to stay at home longer (Digital technology essentials guide [Internet] 2012).

Technology places the patient at the centre of their care, builds a platform for communication between the patient and the service and it enables pathway redesign by utilising the power of IT to re-engineer the outdated process.

Technology can help us deliver high value care as well as target the triple aim of healthcare: the simultaneous improvement of population health, improvement in patient experience of care and reduction in per capita cost. It achieves these through improved lifestyle support and better outcomes across all patient groups, more personalised patient-centred care and journey, and more effective allocation of resources to low-, medium- and high-risk patients respectively.

## Conclusions

In summary, although all VHT have their benefits and limitations, current literature, the results from this study and technology trends within society highlight both M-health and E-forms as the 2 most promising VHT modalities for use in the pre-operative pathway for orthopaedics and hence, the basis of the proposed recommendations.

Developments in technology have over time resulted in many of the VHT uses being available on a single M-health platform, supporting its future use-case. E-forms provide a promising platform for not only information collection and more integrated care, but also an opportunity for the collection of a wealth of electronic data which can be leveraged in the growing domain of healthcare analytics.

In regards to the remaining VHT, website-based information is currently being used and has been deemed ineffective for personalised care, whilst tele-consultations and VR have mixed reviews due to cost. Remote monitoring has previously been proven to be cost-ineffective in major government trials.

### Areas of future research

Possible future areas of research include looking into the barriers of implementation of VHT, as well as possibly exploring patient’s views on their use in the pre-operative pathway. More scope is required to explore the uses of VHT in various other specialties as well as the post-operative pathway. Finally, research is required into the cost effectiveness of technological interventions in the pre-operative pathway. With the literature on this topic increasing exponentially over time, it is crucial to keep tabs on it to ensure application of all the benefits that technology can deliver.

### Limitations of research

Qualitative research methods including the use of SSIs are often criticized as they are limited to the skill of the interviewer, and can be subject to various forms of bias when selecting participants and conducting the interview (Kim et al. 2016; Holloway 2005). Saturation point was judged by the interviewers own personal assessment which may not have been accurate.

As well as this, considering interviews were about asking staff how technology could optimize the pre-operative pathway, the ‘resistance to change’ mentality in the NHS (Anderson 2010) may have had an influence as the HCPs may either not want change or radical change.

Finally, the limitations of TA are often viewed in relation to the flexible nature by which codes are identified and subsequent identification of themes. This often involves personal judgement by those coding the data, which is subject to interpretation bias and inconsistency (Plamping 1998).

## Supplementary Information

**Additional file 1.**

## Data Availability

Please contact author for data requests.

## Abbreviations

E-forms: Electronic forms
GP: General practitioner
HCP: Healthcare professional
M-Health: Mobile health
NHS: National Health Service
OT: Occupational therapist
RPM: Remote patient monitoring
SSI: Semi-structured interview
TA: Thematic analysis
VHT: Virtual healthcare technology
VR: Virtual reality
