# Supplementary figures and images for: Can technology optimise the pre-operative pathway for elective hip and knee replacement surgery: a qualitative study
Source: Perioper Med (Lond). 2020 Nov 16;9:33. doi: 10.1186/s13741-020-00166-0 (PMC7667783; doi:10.1186/s13741-020-00166-0)

Additional file 1


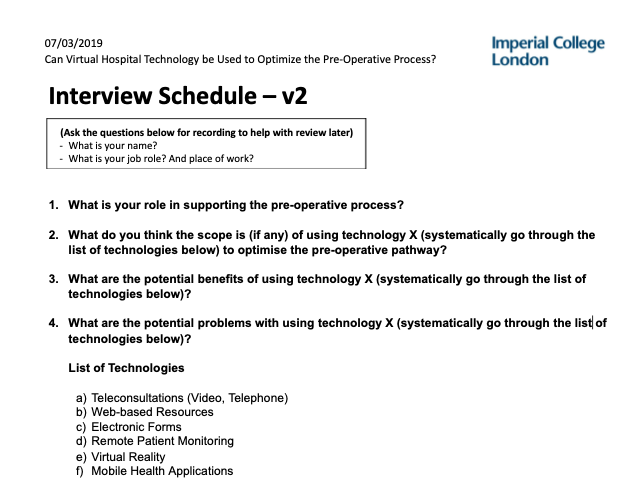

Supplement: Supplementary file 1 — Additional file 1. [file 13741_2020_166_MOESM1_ESM.docx]
